# Lateral ordering of PTCDA on the clean and the oxygen pre-covered Cu(100) surface investigated by scanning tunneling microscopy and low energy electron diffraction

**DOI:** 10.3762/bjoc.10.213

**Published:** 2014-09-01

**Authors:** Stefan Gärtner, Benjamin Fiedler, Oliver Bauer, Antonela Marele, Moritz M Sokolowski

**Affiliations:** 1 Institut für Physikalische und Theoretische Chemie der Universität Bonn, Wegelerstrasse 12, 53115 Bonn, Germany; 2 Present address: Light Technology Institute, Karlsruhe Institute of Technology (KIT), Engesserstr.13, 76131 Karlsruhe, Germany; 3 Departamento de Física de la Materia Condensada, Universidad Autónoma de Madrid, 28049, Madrid, Spain

**Keywords:** Cu(100), LEED, PTCDA, STM, template, thin organic films

## Abstract

We have investigated the adsorption of perylene-3,4,9,10-tetracarboxylic acid dianhydride (PTCDA) on the clean and on the oxygen pre-covered Cu(100) surface [referred to as (√2 × 2√2)*R*45° – 2O/Cu(100)] by scanning tunneling microscopy (STM) and low energy electron diffraction (LEED). Our results confirm the (4√2 × 5√2)*R*45° superstructure of PTCDA/Cu(100) reported by A. Schmidt et al. [*J. Phys. Chem.* **1995,** *99,*11770–11779]. However, contrary to Schmidt et al., we have no indication for a dissociation of the PTCDA upon adsorption, and we propose a detailed structure model with two intact PTCDA molecules within the unit cell. Domains of high lateral order are obtained, if the deposition is performed at 400 K. For deposition at room temperature, a significant density of nucleation defects is found pointing to a strong interaction of PTCDA with Cu(100). Quite differently, after preadsorption of oxygen and formation of the (√2 × 2√2)*R*45° – 2O/Cu(100) superstructure on Cu(100), PTCDA forms an incommensurate monolayer with a structure that corresponds well to that of PTCDA bulk lattice planes.

## Introduction

Interfaces between organic semiconductors and metals have gained large interest because they are present in many applications, e.g., organic field-effect transistors [1] or organic light-emitting diodes [2]. In order to obtain a fundamental understanding of the interactions at these interfaces, the adsorption of perylene-3,4,9,10-tetracarboxylic acid dianhydride (PTCDA) on single crystal surfaces of noble metals has been studied intensively as a model system [3]. On the coinage metal surfaces, the interaction strength of PTCDA increases from physisorption to stronger chemisorption from Au over Ag to Cu. On the (111) surfaces, this is reflected by the decreasing adsorption heights [4–6] and the increasing charge transfer from the metal into the former lowest unoccupied molecular orbital (LUMO) of the PTCDA molecule [7].

For the more open and hence, with respect to the Cu(111) surface, more reactive Cu(100) surface two experimental investigations have been reported [8–9]. Interestingly, they both proposed a dissociative adsorption of PTCDA [8–9] which appears plausible in view of the surface reactivity of Cu(100). However, the two experiments came to slightly different conclusions concerning the dissociation: Schmidt et al. interpret their X-ray photoelectron spectroscopy (XPS) and thermal programed desorption (TPD) data by a loss of the two anhydride O atoms of PTCDA upon adsorption [8]. Andreasson et al. supposed, on the basis of ultraviolet photoelectron spectroscopy (UPS) data, a molecular species after adsorption on Cu(100) which has lost all O atoms, i.e., both the four carboxylic and the two anhydride O atoms [9]. In addition, the two publications reported different superstructures of PTCDA on Cu(100). Schmidt et al. found a (4√2 × 5√2)*R*45° superstructure [8], whereas Andreasson et al. reported a (4.5 × 4.5) structure [9]. The smaller unit cell of the latter fits to the proposed loss of all O atoms. Both structures were suggested on the basis of low energy electron diffraction (LEED) measurements. Scanning tunneling microscopy (STM) data has not been reported yet.

Therefore, the aim of our combined LEED and STM investigation was twofold: on the one hand to clarify the superstructure of PTCDA on Cu(100), and on the other hand to gain new information about the possible dissociation of PTCDA upon adsorption on Cu(100). In addition, we adsorbed PTCDA on a Cu(100) surface that was passivated by preadsorbing oxygen in order to test the impact of the change in the interfacial interaction on the lateral ordering.

## Experimental

The experiments were carried out under ultrahigh vacuum (UHV) conditions. The UHV apparatus consisted of two chambers operated at a base pressure of about 2 × 10^−10^ mbar. The first chamber was used for the preparation of the sample. The cleaning procedure for the Cu(100) crystal consisted of several cycles of sputtering with Ar^+^ ions at an energy of 1000 eV and at a sample temperature of 620 K for 30 min and subsequent annealing at 770 K for 60 min. PTCDA was purchased from Sigma-Aldrich and purified by gradient sublimation. The deposition was performed at sample temperatures of 300–450 K, using a rate of 0.2 monolayers min^−1^. The coverages (θ) were determined from the evaluation of STM images, and in the following one monolayer (1 ML) refers to a coverage equivalent to that of the ordered first layer. The preparation chamber was also equipped with a microchannel plate low energy electron diffraction apparatus (MCP-LEED) from OCI Vacuum Microengineering. The LEED measurements were done at sample temperatures of 280–400 K using beam currents of 1–20 nA. The distortions of the LEED images due to the planar microchannel plate were corrected by a home-written software [10].

The second chamber contained a beetle-type variable temperature STM (VT STM) from RHK technology. All STM images were recorded in constant current mode with a self-cut Pt/Ir tip at room temperature (rt). The given bias voltage *U*_bias_ refers to the sample. After recording the images were treated with histogram alignment (which optimizes the images by inducing an offset for every scan line) and moderate smoothing. For accurate length determination, the STM scanner was calibrated from images of the bare Cu(100) surface. The lengths of the adsorbate unit cell vectors (*a*_0_ and *b*_0_) were determined from STM images as follows. In order to minimize errors from thermal drift only those images were used where the fast and more robust scan direction was parallel to the direction of the considered unit cell vector within ±5°. The angle γ_0_ between the unit cell vectors **a_0_** and **b_0_** was derived from the relative rotation of the fast scan direction that was needed to align it with one or the other unit cell vector. The reported values and errors of the unit cell parameters were computed by statistical averaging of the measured values. The respective errors are the standard deviations plus errors related to uncertainty of the calibration factors of the STM scanner. Other systematic errors are difficult to quantify and are therefore not specified.

To prepare the oxygen covered Cu(100) surface we used a procedure similar to the one reported in [11]. The clean crystal was exposed to an oxygen pressure of 1 × 10^−6^ mbar for 8 min at a temperature of 470 K. Thereafter an annealing step at 700 K for 10 min was performed. Deposition of PTCDA on this oxygen modified Cu(100) surface was carried out at a sample temperature of 300 K with a similar rate as above.

## Results

### PTCDA/Cu(100)

#### Ordering for deposition at 400 K

The structure described in the following section was observed after deposition of about 0.5 ML PTCDA at an elevated sample temperature of 400 K. The dependence of the structure on the sample temperature during deposition and the coverage will be described later.

**Figure 1:**
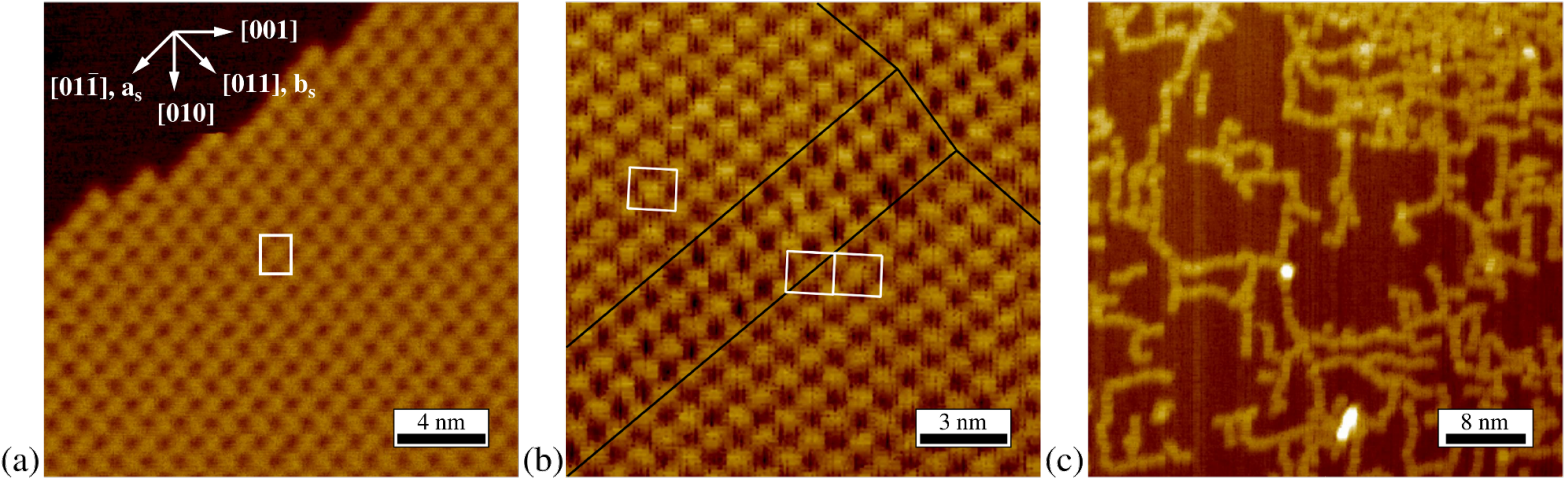
Different STM images of PTCDA on Cu(100). (a) Ordered structure observed after deposition at a sample temperature of 400 K. The upper left corner shows the bare Cu(100) surface. The unit cell of the structure is indicated (*U*_bias_ = −1.2 V, *I*_T_ = 68 pA, 21.6 × 21.6 nm^2^, θ ≈ 0.5 ML). (b) Misfit area between two translational domains. The black lines are guide lines for the eye. The unit cells are indicated (*U*_bias_ = −0.8 V, *I*_T_ = 63 pA, 16.3 × 16.3 nm^2^). (c) PTCDA agglomerates after annealing at 550 K for 5 min (*U*_bias_ = −1.9 V, *I*_T_ = 91 pA, 47.5 × 47.5 nm^2^).

As illustrated by the STM image in Figure 1(a), PTCDA forms islands of an ordered structure on Cu(100). The island edges are often parallel to the diagonal of the unit cell, as visible in the upper left part of Figure 1(a). The structure can be described by a rectangular unit cell, marked in white in Figure 1(a), which contains two differently orientated PTCDA molecules (see also Figure 3(a), below). All parameters measured from STM images of this superstructure are given in the second column of Table 1. Within the error bars we find that the unit cell vectors of the adsorbate structure **a_0_** and **b_0_** are parallel to the [001] and the [010] direction of Cu(100), respectively. This is deduced from the angle φ_0_ between **a_0_** and the substrate vector **a_s_** and the angle γ_0_ between **a_0_** and **b_0_** (see also Figure 3(b), below). Due to the different lengths of **a_0_** and **b_0_**, two rotational domains of the PTCDA structure on Cu(100) are possible. For comparison the unit cell parameters of the commensurate (4√2 × 5√2)*R*45° superstructure reported by Schmidt et al. [8] are given in the first column of Table 1. They agree very well with those determined by us from STM data.

**Table 1:** Overview on the unit cell parameters of structures of PTCDA on Cu(100) (column two) and on the (√2 × 2√2)*R*45° – 2O/Cu(100) surface (column three). For PTCDA/Cu(100) the parameters calculated for the commensurate (4√2 × 5√2)*R*45° structure derived by Schmidt et al. [8] are compared to those we derived from our STM data. γ_0_ denotes the angle between the unit cell vectors **a_0_** and **b_0_**, the lengths are *a*_0_ and *b*_0_, respectively. φ_0_ denotes the angle between **a_0_** and the vector **a_s_** of the unit cell of the Cu(100) surface along the 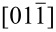 direction (see Figure 4(d)). The last row of the table gives the superstructure matrices **M**_0_ with respect to the substrate vectors of Cu(100).

	PTCDA/Cu(100)		PTCDA/O/Cu(100)

	(4√2 × 5√2)*R*45° [8]	Own STM results	STM results

*a*_0_ [Å]	14.46	14.5 ± 0.3	12.2 ± 0.3
*b*_0_ [Å]	18.08	18.0 ± 0.4	19.3 ± 0.4
γ_0_ [°]	90	89.9 ± 0.8	89.3 ± 1.2
φ_0_ [°]	45	45.2 ± 0.8	38.8 ± 0.8
**M**_0_	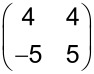	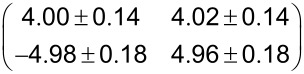	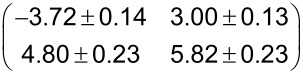

**Figure 2:**
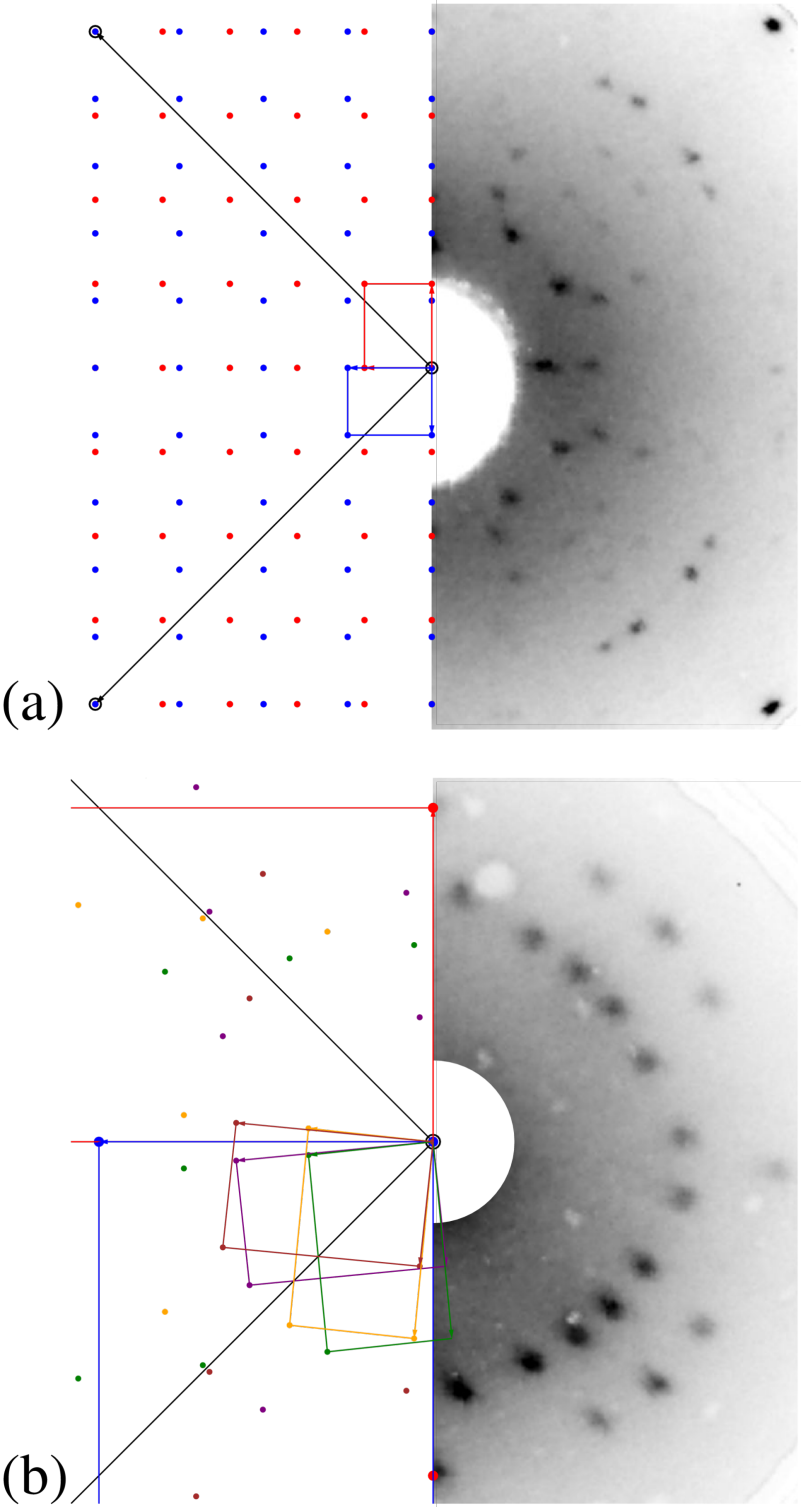
(a) LEED pattern of PTCDA on Cu(100) observed after deposition at a sample temperature of 400 K (62.5 eV, θ ≈ 0.5 ML). Left hand side: Corresponding calculated LEED pattern. The reciprocal unit cells of the Cu(100) surface (black) and of the two symmetry equivalent domains of the commensurate (4√2 × 5√2)*R*45° superstructure (blue and red) are indicated. (b) LEED pattern of PTCDA on (√2 × 2√2)*R*45° – 2O/Cu(100) (16.6 eV, θ ≈ 0.5 ML). Left hand side: Corresponding calculated LEED pattern. The reciprocal unit cells of the (√2 × 2√2)*R*45° – 2O/Cu(100) (blue and red) and the four symmetry equivalent domains of the incommensurate superstructure of PTCDA according to the matrix **M**_0_ (see third column of Table 1) are indicated. Note that the unit cells of the (√2 × 2√2)*R*45° – 2O/Cu(100) surface do not fit completely into the depicted area of the reciprocal space.

The right part of Figure 2(a) shows the LEED pattern of the PTCDA/Cu(100) including the first order spots of the substrate. A simulation of the LEED pattern is shown on the left. The substrate spots are drawn in black; the two domains of the (4√2 × 5√2)*R*45° superstructure are drawn in red and blue. The simulated LEED pattern explains well the experimental LEED pattern. We note that in this LEED pattern and in those observed at higher electron energies, superstructure spots of high order are well visible. This indicates a commensurate superstructure, because in this case multiple scattering from the substrate can contribute to the superstructure spots, thus increasing the intensities of the LEED spots at higher orders. For a commensurate structure, a PTCDA induced buckling of the Cu(100) surface is very conceivable. This can also contribute to the intensities of the superstructure LEED spots. We note that a significant surface buckling was recently derived from density functional theory calculations for PTCDA on Ag(111), Ag(100) and Ag(110) [12]. Therefore it seems reasonable to assume a PTCDA induced buckling on Cu(100), too.

From LEED patterns at lower electron energies we further find that the (1,0) and (3,0) spots are not visible. This indicates a systematic extinction for the (*n*,0) and (0,*n*) spots with *n =* odd which is due to a *p2gg* symmetry of the structure (i.e., the presence of two perpendicular glide planes). It was already noted by Schmidt et al. [8] and related to a *p2gg* symmetry of the PTCDA layer. As we will describe below, application of this symmetry to the entire adsorption complex (including the Cu(100) substrate) restricts the possible adsorption sites of PTCDA.

The commensurate matrix **M**_0_ of the (4√2 × 5√2)*R*45° superstructure corresponds to the observed LEED pattern, and within the error bars, **M**_0_ is identical with the matrix we calculated from the STM measurements (see Table 1). Therefore our data obtained for deposition of PTCDA on Cu(100) at an elevated sample temperature of 400 K confirm the structure proposed by Schmidt et al. [8]. We did not observe the structure proposed by Andreasson et al. [9].

#### Kinetic hindrance for deposition at 300 K

Depending on the sample temperature during the deposition the fraction of (4√2 × 5√2)*R*45° ordered regions on the covered surface varies. For lower deposition temperatures (300–350 K) we find only small domains of typically less than 50 Å in diameter which are separated by misfit areas between them. In these misfit areas, the structure slightly deviates from the (4√2 × 5√2)*R*45° superstructure. Figure 1(b) shows such a misfit area between two translational domains of the superstructure. We use the term misfit area instead of the usually used term domain boundaries in order to emphasize that these areas have a certain width and span several unit cells. The structure of the misfit areas exhibits some flexibility and varies depending on the relative positions of the (4√2 × 5√2)*R*45° domains which are connected. Apparently, at lower deposition temperatures the surface mobility of the PTCDA molecules is too small to allow the structural adaptation of different translational or rotational domains which have resulted from incoherent nucleation. This causes a significant fraction of the surface to be covered by misfit areas.

In the same way as the edges of islands, the borders between ordered domains and misfit areas are also often orientated parallel to the diagonal of the adsorbate unit cell. Due to the misfit areas LEED patterns of samples prepared at low temperatures show only broad and weak spots. From STM images we find that for a deposition temperature of 300 K only about 65% of the covered surface is ordered in the (4√2 × 5√2)*R*45° superstructure, whereas this fraction increases to 90% for a deposition temperature of 400 K. In each case, the rest of the surface is covered by misfit areas. In addition to the decreasing fraction of the misfit areas, the average domain size increases for higher deposition temperatures. Whilst for a deposition temperature of 300 K, the average domain size is only about 50 nm^2^, deposition at 400 K leads to an average size of about 150 nm². LEED images show that post annealing at 450 K for 10 min of a sample prepared at a temperature of 300 K transforms the misfit areas into ordered domains of the (4√2 × 5√2)*R*45° superstructure. Before the post annealing, closely adjacent LEED spots could not be distinguished due to their broadness, whereas this was well possible afterwards. The resulting LEED pattern is identical to the one of a sample prepared at 400 K. However, further annealing at 550 K for 5 min leads to a complete destruction of the ordered superstructure. In the LEED pattern the adsorbate spots have vanished then, and STM images show only chain-like disordered structures, as illustrated in Figure 1(c), which will be discussed further below in the Discussion section.

In contrast to the temperature, a variation of the coverage has no strong influence on the quality of the superstructure. Finally, our LEED and STM data (not shown) confirm that even the second layer of PTCDA orders according to the (4√2 × 5√2)*R*45° superstructure, as it was already proposed by Schmidt et al. [8].

#### The adsorption site

**Figure 3:**
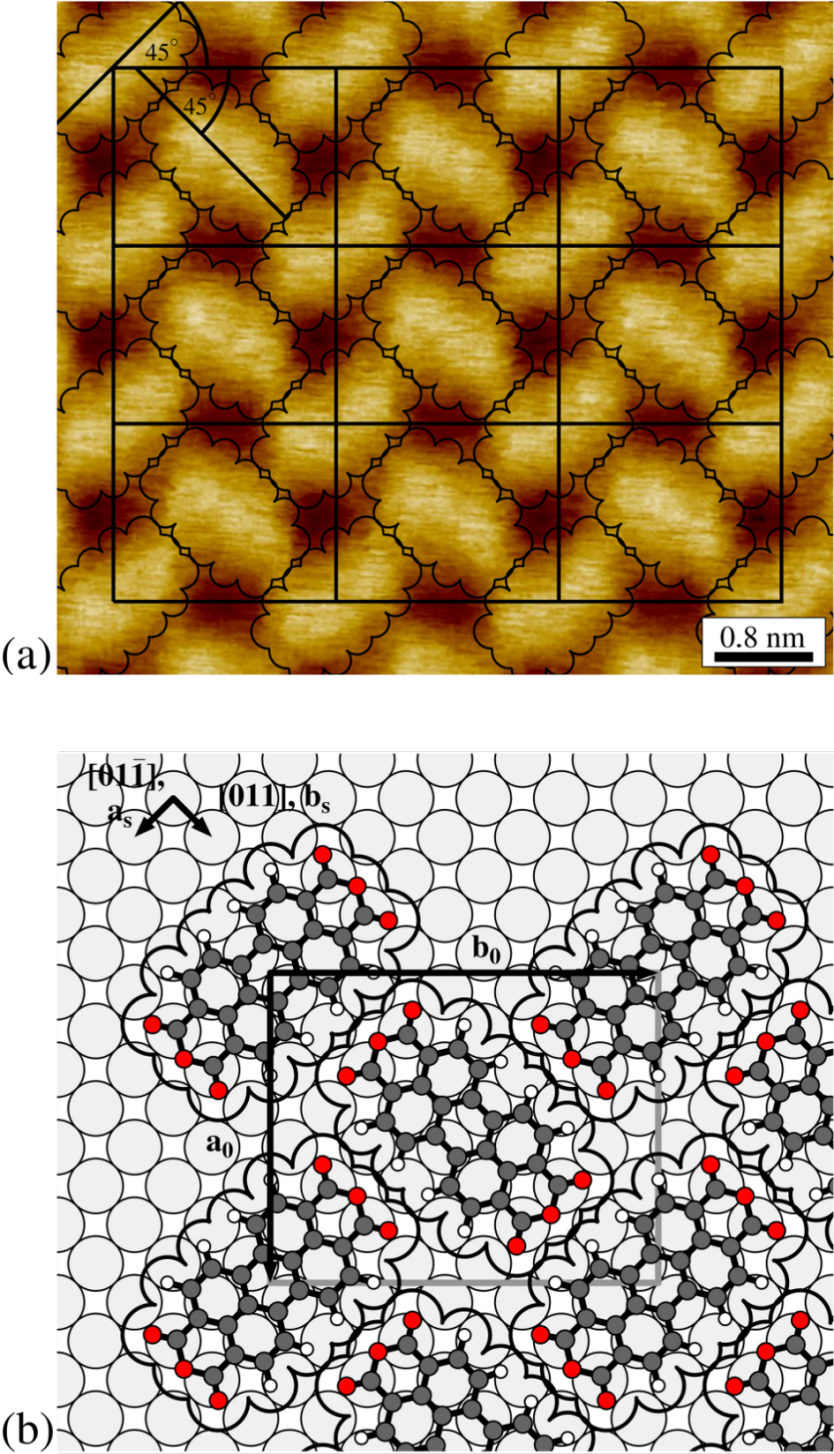
(a) High resolution STM image of the (4√2 × 5√2)*R*45° structure of PTCDA on Cu(100). Here the influence of the thermal drift has been corrected (*U*_bias_ = 1.3 V, *I*_T_ = 67 pA, 6.3 × 5.5 nm^2^). 3 × 3 unit cells and the van der Waals radii [13] of the PTCDA molecules are indicated (black lines). (b) Hardsphere model for the (4√2 × 5√2)*R*45° structure of PTCDA on Cu(100). Carbon atoms: dark grey, oxygen atoms: red, and hydrogen atoms: white, copper atoms light grey. Further details see text.

We now turn to a more detailed analysis of the packing within the unit cell. Figure 3(a) shows a close up view of the (4√2 × 5√2)*R*45° superstructure of PTCDA on Cu(100). The influence of the thermal drift has been corrected by determination of the unit cell in the uncorrected image and setting its parameters to the commensurate ones. In order to determine the orientation of the two molecules within the unit cell the outer contours of the molecules given by the van der Waals spheres [13] were superimposed and fitted to the STM image. With an estimated uncertainty of about ±3° the long molecular axes are orientated 45° with respect to the unit cell vectors. Therefore the two PTCDA molecules within each cell are positioned perpendicular to each other in an L-shape arrangement and their long molecular axes are parallel to the 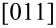 and the 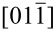 directions of Cu(100), respectively.

As visible in Figure 3(a), the STM contrast of the two molecules within the unit cell is identical and independent on the orientation of the molecule. This observation was also made for STM images taken at other bias voltages than 1.3 V (used for Figure 3(a)), namely of −1.2 V, −0.55 V, and 0.86 V. Hence we propose that both molecules exhibit identical adsorption sites. This conclusion is based on the observation of a significantly different STM contrast for structures where two PTCDA molecules are on different adsorption sites in the unit cell, e.g., for PTCDA on Ag(111) [14]. The difference in the STM contrast is a consequence of the different electronic coupling of the molecules to the substrate on the two different sites. We note however that the observation of the STM contrast is not a firm argument for identical adsorption sites and may require validation by other methods.

Under the condition that one PTCDA molecule is positioned at the edge and one at the center of the unit cell, as it is derived from the STM images, two identical adsorption sites are only possible, if the centers of the two molecules are positioned on bridge sites of the Cu(100) surface (see Figure 3(b)). Since the (100) surface exhibits two different bridge sites, rotated by 90° to each other, two possibilities exist for positioning the adsorbate structure on Cu(100).

For both possibilities the whole adsorbate/substrate complex exhibits all symmetry elements which are required for the *p2gg* symmetry of the (4√2 × 5√2)*R*45° superstructure derived from LEED, and in both cases the application of all symmetry elements of the substrate leads to two rotational domains, in agreement with the experimental findings. We cannot exclude one of the two possibilities on the basis of our data so far. However we propose that the structure imaged in Figure 3(b), is favorable because in this structure the carbon atoms of the anhydride groups (C_func_) come on on-top positions, which would be not the case for the alternative structure. A side-to-side illustration of both possibilities is given in Supporting Information File 1. We note that due to the different lattice constant of Cu compared to Ag, an arrangement of all carboxylic O atoms close to on-top sites, as it is found for PTCDA on Ag(100) [12] is not possible. Hence, the positioning of the C_func_ atoms on top-positions may act as an alternative surface bonding motive here. In the model proposed by Schmidt et al. [8], the PTCDA molecules exhibit two different adsorption sites: The center of one molecule is positioned on a four-fold hollow site, whereas the other one is positioned on an on-top site. This model would hence contradict our observation of an identical STM contrast for both molecules in the unit cell. In addition, the adsorbate/substrate complex of the model proposed by Schmidt et al. has a reduced symmetry compared to the adsorbate layer alone and we expect that the extinctions in the LEED pattern due the *p2gg* symmetry would be lifted, similar to the situation seen for PTCDA/Ag(111) [14].

#### PTCDA in the oxygen pre-covered Cu(100) surface

**Figure 4:**
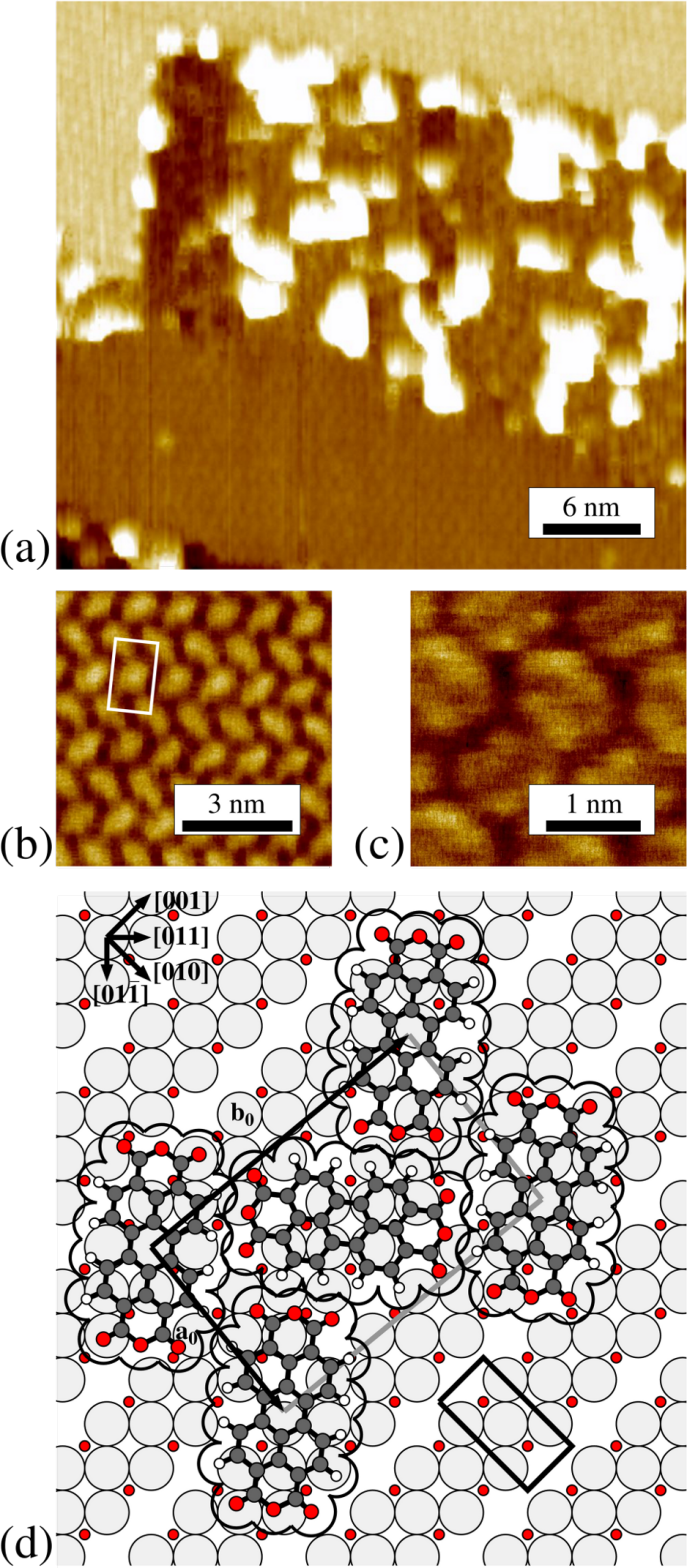
Different STM images of PTCDA on the (√2 × 2√2)*R*45° – 2O/Cu(100) surface. (a) STM image of a terrace, which is fully covered by PTCDA. The terrace is limited by an ascending and a descending step in the upper and lower parts of the image, respectively (*U*_bias_ = −3.0 V, *I*_T_ = 49 pA, 38.8 × 35.0 nm^2^). (b) Image of the ordered incommensurate structure of PTCDA on this surface (*U*_bias_ = −1.6 V, *I*_T_ = 59 pA, 8.0 × 8.0 nm^2^). (c) High resolution image of the ordered structure showing submolecular resolution (*U*_bias_ = 1.2 V, *I*_T_ = 84 pA, 3.1 × 3.1 nm^2^). (d) Hardsphere model for the incommensurate structure of PTCDA on (√2 × 2√2)*R*45° – 2O/Cu(100). The unit cell of the 2O/Cu(100) surface is drawn in the right lower corner. In order to reduce the complexity only the topmost layer of one domain of the (√2 × 2√2)*R*45° – 2O superstructure is drawn. The same color code as in Figure 3 applies.

The adsorption of atomic oxygen on Cu(100) induces a missing row reconstruction where every fourth 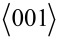 row of copper atoms is missing, leading to a (√2 × 2√2)*R*45° – 2O/Cu(100) superstructure [15]. This reconstruction is illustrated in Figure 4(d) (below). The structural details have been taken from [15]. The corresponding unit cell, containing two oxygen atoms, is indicated in lower right corner of Figure 4(d). We note that the structural description has been discussed in the literature for some time concluding in this missing row reconstruction (see references in [15]). Our LEED and STM measurements confirm that the preparation procedure described above leads to the formation of this (√2 × 2√2)*R*45° – 2O/Cu(100) superstructure with two rotational domains.

PTCDA forms large, nearly defect-free domains on the (√2 × 2√2)R45° – 2O/Cu(100) surface, already at a deposition temperature of 300 K. Figure 4(a) shows an STM image of a terrace with a nominal coverage of 1 ML of PTCDA. The terrace is limited by a descending and an ascending step. Interestingly, the ordered PTCDA structure is only present in the region adjacent to the descending step (see lower left corner of Figure 4(a)). Further away from the step, a disordered structure with bright protrusions appears, extending to the ascending step. This type of disordered structure at the lower step edge sites is also present at low coverages. We propose that it may be possible that individual Cu atoms get pulled out of the step edges here and interact more strongly with the PTCDA molecules closer to the steps, leading to the formation of these clusters. Presumably the effect is promoted by the presence of the oxygen induced reconstruction and hence only present on the O pre-covered Cu(100) surface, and not on the bare Cu(100) surface.

The step edges of the PTCDA covered surface are facetted and the straight sections run parallel to the [001] and [010] directions. The steps are hence orientated parallel or perpendicular to the missing rows of the (√2 × 2√2)*R*45° – 2O/Cu(100) surface, as already observed for the uncovered 2O/Cu(100) surface. We take this as an indication that the (√2 × 2√2)*R*45° – 2O/Cu(100) superstructure remains unchanged underneath the PTCDA adsorbate layer. This is also confirmed by LEED patterns of PTCDA on 2O/Cu(100) which show the reconstruction spots, although with damped intensities. Because of the small size of the domains of the 2O/Cu(100) reconstruction of less than 50 nm^2^ (in agreement with [16]) the PTCDA superstructure overgrows the domain boundaries of the 2O/Cu(100) surface.

A close up of the ordered PTCDA structure is shown in Figure 4(b). The unit cell (marked in white) contains two PTCDA molecules with different orientations. In contrast to the superstructure of PTCDA on bare Cu(100), the longer vector **b_0_** of the unit cell forms an angle of about 6° with respect to the [001] direction of (100) surface and also the dimensions of the unit cell vectors differ with respect to those of PTCDA on bare Cu(100). We note that contrary to the situation on bare Cu(100) we get a more pronounced submolecular contrast of the PTCDA for similar tunneling parameters (see Figure 4(c) in comparison with Figure 3(a)) which we interpret as an indication for the electronic decoupling from the substrate. The structural parameters derived by STM are given in Table 1. The corresponding superstructure is incommensurate with respect to the bare Cu(100) as well as to the 2O/Cu(100) surface. This indicates a weak interaction of the PTCDA with the surface. The diffraction pattern of the PTCDA superstructure is simulated in the left part of Figure 2(b) and compared with the observed LEED pattern, which is displayed in the right part. The direction of the substrate vectors are indicated by black lines. The unit cells of the two rotational domains of the underlying (√2 × 2√2)*R*45° – 2O reconstruction are indicated in red and blue. The remaining spots are all explained well by four symmetry equivalent domains of the PTCDA superstructure, consisting two mirror domains of the PTCDA structure on both of the two rotational domains of the 2O/Cu(100) surface. The respective unit cells are indicated in Figure 2(b). Notably, the (1,0) and the (0,1) LEED spots of the PTCDA layer cannot be detected. We explain this extinction with the *p2gg* symmetry of the PTCDA layer. Due to its incommensurability with the 2O/Cu(100) substrate, the scattering of the substrate does not contribute to the LEED spots of the PTCDA layer and hence does not have an effect on the extinction of the (1,0) and (0,1) spots. This is different from the situation discussed above for the commensurate structures of PTCDA on Cu(100) or Ag(111), where the substrate can lift the extinction rule.

As noted, the four symmetry equivalent domains of PTCDA consist of two pairs of mirror domains. A structure model of one domain is shown in Figure 4(d). (We note that we have chosen the domain, which fulfills the requirements of naming superstructures defined by Barlow and Raval [17].) A symmetry equivalent domain is obtained by a mirror operation with respect to the [001] direction or the [010] direction, respectively. The second pair of domains is then obtained by a 90° rotation of the underlying (√2 × 2√2)*R*45° – 2O superstructure, yielding its symmetry equivalent rotational domain. However, we propose that all four symmetry equivalent PTCDA domains exist on both rotational domains of the (√2 × 2√2)*R*45° – 2O/Cu(100) structure, because the PTCDA superstructure overgrows the respective domain boundaries (see above).

## Discussion

### Structural considerations

It is interesting to compare the lateral ordering within the commensurate (4√2 × 5√2)*R*45° superstructure of PTCDA on Cu(100) with that within the commensurate (4√2 × 4√2)*R*45° superstructure of PTCDA on Ag(100) [18]. Both structures contain two PTCDA molecules per unit cell, in perpendicular orientation to each other, motivated by the electrostatic interaction between the partially negatively charged anhydride groups and the positively charged perylene cores. On Cu(100) the area per molecule corresponds to 131.2 Å^2^. This value is very comparable to that for PTCDA on Ag(100) (132.9 Å^2^). Both values are about 10% larger than the area per molecule in the lattice planes of PTCDA bulk crystals of the α or β-modification (120 Å^2^) [19]. Interestingly the unit cell on Cu(100) is rectangular, while it is quadratic on Ag(100). We propose that this is mainly a consequence of the smaller lattice constant of Cu with respect to Ag in combination with a strong chemisorptive interaction of PTCDA with the substrate, leading to a preferred azimuthal orientation of the long molecular axes along the 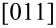 and the 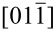 directions.

The bonding of the PTCDA molecule to the Cu(100) substrate is of course most important for the structure formation. It causes the preferential orientation of the molecular axes along the 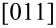 and the 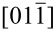 directions, i.e., the close packed rows of the substrate, because the resulting high symmetry of the adsorbate complex presumably optimizes the molecule/substrate interactions. On Cu(100), the two hypothetic commensurate structures with a quadratic unit cell, which yield this orientation of the molecules along the 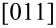 and the 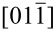 directions, are either too large ((5√2 × 5√2)*R*45° /163.8 Å^2^ per molecule) or too small ((4√2 × 4√2)*R*45° /105.1 Å^2^ per molecule). Other quadratic commensurate superstructures on Cu(100) with a similar size to that of PTCDA/Ag(100) are rotated with respect to the former. Such a structure is, e.g., 
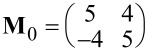
 with 134.5 Å^2^ per molecule and φ_0_ = 38.7°. However, under the assumption that the favorable orientation of the long molecular axes along the 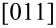 and the 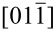 directions was maintained in this structure, the spaces between the anhydride groups would be too small, which is unfavorable due to the negative partial charges on these groups. We note that we actually found this superstructure as a minor phase at the beginning of our experiments due to a (presumably partially positively charged) contamination, e.g., carbon, which stabilized the closeness of the anhydride groups. Due to an increasing cleanliness of the sample during the following experiments this structure disappeared. In conclusion, on Cu(100), a rectangular unit cell is preferred because this cell yields orientations of the long molecular axes along the 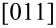 and the 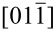 directions and simultaneously optimizes the intermolecular interaction of the negatively charged anhydride groups with the positively charged perylene cores.

### Bonding and dissociation

We suppose that UPS will reveal a chemisorptive bonding of PTCDA on Cu(100) with filling of the former LUMO, similar to the situation that was found on Ag(100) [20]. We note that the UPS measurements from Andreasson et al. of PTCDA on Cu(100) can also be interpreted in this way [9], although these results have to be taken with some caution, because of the different structure they found. The ordering of the second layer of PTCDA on Cu(100) with the same commensurate structure as the monolayer further points to a strong interaction with the Cu substrate. This is in contrast to the formation of an incommensurate second layer of PTCDA on Ag(100) [18]. The formation of kinetically stabilized misfit areas at a deposition temperature of 300 K on Cu(100) is also indicative for the strong interfacial interaction on Cu(100). On Ag(100), PTCDA forms highly ordered domains already at this temperature [18]. Therefore the corrugation of the bonding potential for PTCDA/Cu(100) seems to be significantly larger compared to PTCDA/Ag(100), which leads to a lower diffusion constant and hence the need of higher preparation temperatures for highly ordered layers.

However, the question whether the chemisorption is dissociative or not still needs to be answered. So far, all our structural findings stand against dissociative adsorption: (a) In STM images, like Figure 3(a), the van der Waals-radii of the PTCDA molecules fit well to the measured electron density, although a loss of only the bridging oxygen atoms could certainly not be recognized. (b) The derived structure can be well understood from arguments based on intact PTCDA molecules, in particular the area per molecule agrees well with the expected one. (c) The structural order in the layer improves upon moderate heating to 400 K. If dissociation took place during the deposition, we would expect that heating would activate the formation of irreversible intermolecular and/or molecule substrate bonds which would counteract the formation of a long range ordered structure.

Data reported in the literature does not give a firm proof of a dissociative adsorption of PTCDA on Cu(100). The XPS measurements by Schmidt et al. were carried out with PTCDA on a polycrystalline copper foil [8]. Therefore the result that the ratio C:O is about 6:1 instead of 4:1 for a low coverage (indicating the presence of only four instead of six oxygen atoms per PTCDA molecule) cannot be easily transferred to the single crystal Cu(100) surface. Schmidt et al. support their conclusion also by TPD experiments of PTCDA on Cu(100) [8], in which they did not observe a peak of the fragment *m*/*z* = 124 for a coverage of 1 ML. This observation could be due to PTCDA dissociation during the adsorption process, but it is also conceivable that the dissociation occurs at elevated temperatures during the TPD experiment itself. This scenario is known from other chemisorptive systems, like PTCDA/Ag(111), [21]. Our STM data support such a scenario also for PTCDA on Cu(100). Figure 1(c) shows the chain like structure observed after annealing the (4√2 × 5√2)*R*45° superstructure for 5 min at 550 K. Some chains are isolated and some chains are packed closer together, probably with remaining PTCDA molecules in between them (see upper right corner of Figure 1(c)). Since there are apparently also chains with threefold connection points, a simple polymeric reaction, which took place at the reactive anhydride groups of the PTCDA molecules can be excluded. However, it is hence feasible that at this elevated temperatures the PTCDA molecules have undergone different kinds of chemical reactions. Therefore no intact desorption can be expected, in agreement with the TPD experiments of Schmidt et al. [8]. A non-dissociative adsorption on Cu(100) is further corroborated by STM images of PTCDA on the even more open, and hence more reactive, Cu(110) surface, where no dissociation after adsorption at rt and post annealing at 450 K was found [22]. In conclusion we state that in spite of the strong interaction between PTCDA and Cu(100) there are no indications for a dissociation during adsorption.

### Surface passivation

The situation changes drastically for the adsorption of PTCDA on the oxygen induced reconstruction of the Cu(100) surface. The structure is incommensurate and has a unit cell of a size of 12.2 × 19.3 Å^2^, which is hence very similar to that of the respective lattice plane in bulk PTCDA crystals of the β-modification. The area per molecule is 117.7 Å^2^ which has to be compared with 120.6 Å^2^ for the β-modification. This indicates a dominant character of the intermolecular interactions in this system. All these observations reveal that for this system the adsorbate/adsorbate interactions have more influence on the structure formation than the adsorbate/substrate interactions, which contrasts with the findings for PTCDA on bare Cu(100), where the latter interactions are dominant.

Thus the (√2 × 2√2)*R*45° – 2O superstructure passivates the reactive Cu(100) surface effectively. A similar phenomena is known for the adsorption of PTCDA on the reactive Ni(111) surface. In that case the interaction on the free surface is so strong that the molecules do not diffuse over the surface and hence do not form an ordered structure [23], whereas on *p*(2 × 2) – O/ /Ni(111) a well ordered herringbone structure was observed [24]. The passivation with an oxygen layer therefore leads to an increase of the diffusion constant. On Cu(100) an elevated surface temperature of 400 K is needed to obtain a highly ordered structure, whereas this is possible for PTCDA on (√2 × 2√2)*R*45° – 2O/Cu(100) already at rt.

## Conclusion

From STM and LEED data we confirm the commensurate (4√2 × 5√2)*R*45° superstructure of PTCDA on Cu(100) proposed by Schmidt et al. [8]. A structure model where both molecules in the unit cell are on identical sites has been derived. On the basis of our structural data, there are no indications for dissociation during the adsorption. However, kinetic barriers hinder the lateral ordering at 300 K, leading to significant misfit areas between the ordered domains. These can be avoided if the preparation is performed at 400 K. STM images show that at 550 K the PTCDA molecules undergo a chemical reaction, which prohibits intact desorption. On the oxygen passivated (√2 × 2√2)*R*45° – 2O/Cu(100) surface, the interfacial interaction is strongly reduced, leading to an ordered PTCDA layer with an incommensurate structure.

## Supporting Information

File 1 Two possible adsorption sites of PTCDA on Cu(100).
