# Supplementary material for: Lateral ordering of PTCDA on the clean and the oxygen pre-covered Cu(100) surface investigated by scanning tunneling microscopy and low energy electron diffraction
Source: Beilstein J Org Chem. 2014 Sep 1;10:2055–64. doi: 10.3762/bjoc.10.213 (PMC4168817; doi:10.3762/bjoc.10.213)

Supporting Information

for

Lateral ordering of PTCDA on the clean and the oxygen pre-covered Cu(100) surface investigated by scanning tunneling microscopy and low energy electron diffraction

Stefan Gärtner[1,2], Benjamin Fiedler[1], Oliver Bauer[1], Antonela Marele[3], and Moritz M. Sokolowski*[1]

Address: Institut für Physikalische und Theoretische Chemie der Universität Bonn, Wegelerstrasse 12, 53115 Bonn, Germany, [2]Present address: Light Technology Institute, Karlsruhe Institute of Technology (KIT), Engesserstr.13, 76131 Karlsruhe, Germany and 3Departamento de Física de la Materia Condensada, Universidad Autónoma de Madrid, 28049, Madrid, Spain.

Email: Moritz M. Sokolowski* - sokolowski@pc.uni-bonn.de

*Corresponding author

Two possible adsorption sites of PTCDA on Cu(100)

Below we illustrate the two possible adsorption sites of PTCDA on Cu(100) that are both compatible with our LEED and STM results. As mentioned in the article, we suggest that the site shown on the left hand side is the correct one, due to the *on-top* positions of the functional C atoms in the anhydride groups. The symmetry elements of the two structures are indicated in addition.

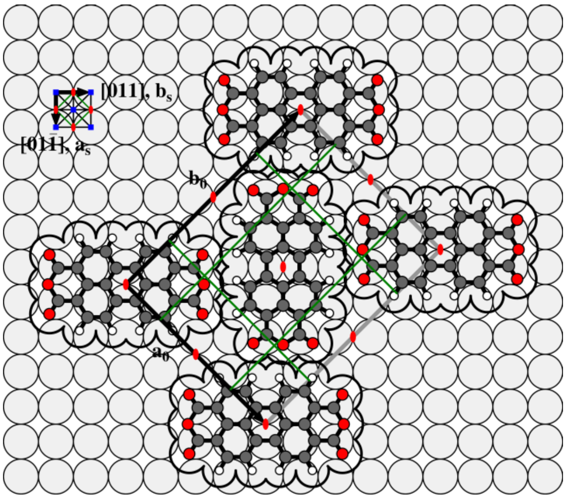

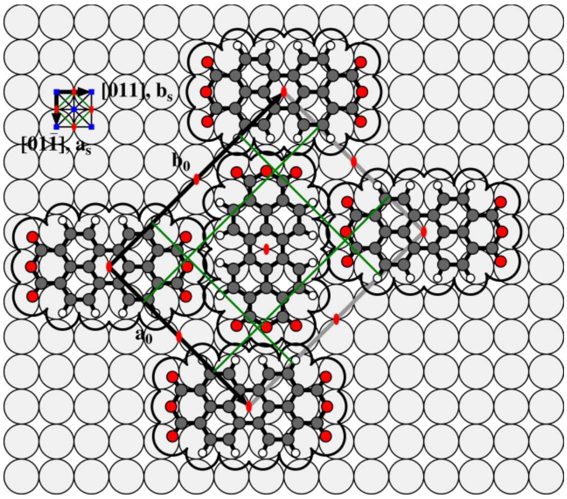

Supplement: File 1 — Two possible adsorption sites of PTCDA on Cu(100). [file Beilstein_J_Org_Chem-10-2055-s001.pdf]
